# Vaccination in the Light of the Royal British Commission

**Published:** 1898-12

**Authors:** M. R. Leverson

**Affiliations:** Fort Hamilton, L. I., N. Y.


### VACCINATION IN THE LIGHT OF THE ROYAL BRITISH COMMISSION.

M. R. Leverson, M. D., Fort Hamilton, L. I., N. Y.

(Continued from Nov. No., page 509.)

At the session of June 25th, 1890, Mr. Tebb continued his testimony. The Commission declined to receive a *letter* from Mr. Bendenell Carter, on the ground that Mr. Carter being still alive, his evidence could be obtained directly from him if necessary.*

Q. 10,001. As to the connection between leprosy and vaccination Mr. Tebb quotes several cases; also the opinion of Dr. J. Bechtinger, of Rio de Janeiro, who has devoted twenty-seven years to the study of leprosy in the West Indies, in the Sandwich Islands, in South America, India, and in British Guiana, who says that "Vaccination is responsible for a serious augmentation of the disease in these countries." He believes this is partly due to syphilitic virus imported from Europe, *which becomes leprous*, and partly from leprous virus used in those countries. He has often been consulted by parents whose families were quite free from any taint of leprosy, which he has traced in the children to vaccination.

Q. 10,002-5. The Commission is surprised at the suggestion that syphilis may become leprosy. Dr. Bechtinger has published an extensive work on leprosy (some 500 pages).

Q. 10,006 (By Professor Michael Foster). "Does he con-

---

* This course was adopted by the Commission in nearly all cases. It was the proper course to take in the case of persons living in Great Britain, but should have been supplemented by taking steps to call before them such persons who were reluctant to attend, if their evidence would be likely to be of use.

An undertaking was given on the part of Sir John Simon that he would appear before the Commission to explain the important concealments of which the records produced by Mr. Tebb accused him. He never did so; but the Commission ought in all fairness to have compelled his appearance.—M. R. L.

sider leprosy a development of syphilis? He considers that under certain conditions syphilis may induce leprosy."

Q. 10,007-8. The witness quotes Dr. Robert Francis Black (M. R. C. S., L. R. C. P., etc), fourteen years a resident of the Port of Spain, who has seen children die of erysipelas from vaccination, but who maintains that the greatest danger in the West Indies was the invaccination of leprosy. In *The British Medical Journal* for June 11th, 1887, is a communication from Dr. W. T. Gairdner, Professor of Medicine in Glasgow University, entitled, "A Remarkable Experience Concerning Leprosy, Involving Certain Facts and Statements Bearing on the Question, 'Is Leprosy Communicable Through Vaccination?' "

Q. 10,009. The narrative of Professor Gairdner detailed the infection with leprosy of two youths by vaccination. Dr. Black reports to the Governor of Trinidad that his experience agrees with that of Professor Gairdner that leprosy has been communicated by vaccination in several cases.

Q. 10,013. A letter from Dr. Gairdner, stating that all the information he possesses upon the subject of invaccinated leprosy has been published by him and is accessible to the Commission, and as he has nothing further to say, it is not his intention to go before the Commission.

Q. 10,014-20. Mr. Tebb then proceeds to detail his own experience. As this is all given, even more fully, in Mr. Tebb's work, *The Recrudescence of Leprosy*, which is readily accessible, it is not deemed necessary to reproduce it. Further, the fact is now admitted by the advocates of vaccination, who make it one of the reasons for abandoning "arm to arm" vaccination and for using the cow-pox virus direct from the calf; thus giving the vaccinees the risks of any bovine disease with which the calf may be endowed. At the same time it is to be observed that the Commission struggled with Mr. Tebb to try and weaken the force of the evidence produced, but without success. At Qs. 10,021-22 Mr.

Tebb quotes the report of the Leprosy Committee of the Royal College of Physicians to the effect that leprosy is not contagious in the conventional sense.

Q. 10,023. Dr. Bristowe dissents from that view.

Q. 10,084. The witness mentions the opinion prevailing among medical men, both in the West Indies and in British Guiana, that leprosy is disseminated by mosquitoes. Mr. Tebb, in his work above cited (p. 129), mentions also China and Japan as countries where that opinion prevails.

Q. 10,088. Mr. Hutchinson says that leprosy has diminished in New Zealand during the vaccination period. Mr. Tebb answers that he understood leprosy was confined to the Maoris, to which Mr. Hutchinson says, "Yes, almost so; and notwithstanding vaccination leprosy has diminished and says that the increase of leprosy is a matter of dispute." Mr. Tebb says, "It may be a matter of dispute here, but it is no matter of dispute in the West Indies. It is a universal belief."

Q. 10,091-2. Mr. Hutchinson says it is increasing in the Sandwich Islands but steadily diminishing in most places, also possibly increasing in the West Indies.

Q. 10,094. Mr. Tebb sums up the result of his inquiries as follows:

1. Evidence from all authorities shows that leprosy is seriously increasing throughout the West Indies and other countries.

2. The theory of contagion put forward to account for this increase is doubtful, is denied by the high medical authorities both at home and abroad, and if true, would only account for an infinitesimal portion of such increase.

3. All authorities admit that leprosy may be communicated by inoculation.

4. That the only method of inoculation extensively practiced is by means of (arm to arm) vaccination, and that leprosy has been distinctly traced to this source by medical practitioners in the West Indies, by several medical superintendents of the leper asylums, and by distinguished authorities, such as Dr. Tilbury Fox, Sir Erasmus Wilson, Dr. Gavin Milroy, Professor W. T. Gairdner, of Glasgow; Dr. Hillis, Dr. Ed. Armix, Dr. Sieving, Dr. A. M. Brown, Dr. Hall Bakewell, Dr. Bechtinger, Sir Morell Mackenzie, and others.

